# A novel mutation alters the stability of PapA2 resulting in the complete abrogation of sulfolipids in clinical mycobacterial strains

**DOI:** 10.1096/fba.2018-00039

**Published:** 2019-04-10

**Authors:** Vipul Panchal, Nidhi Jatana, Anchal Malik, Bhupesh Taneja, Ravikant Pal, Apoorva Bhatt, Gurdyal S Besra, Lipi Thukral, Sarika Chaudhary, Vivek Rao

**Affiliations:** ^1^ Cardio Respiratory Disease Biology CSIR‐Institute of Genomics and Integrative Biology New Delhi India; ^2^ Academy of Scientific and Innovative Research, CSIR- Human Resource Development Centre (CSIR-HRDC) Campus New Delhi India; ^3^ National Institute of Immunology New Delhi India; ^4^ School of Biosciences and Institute of Microbiology and Infection University of Birmingham Birmingham UK

**Keywords:** Mycobacterium tuberculosis, Mtb lineages, Cell wall lipids, PapA2, Sulfolipids

## Abstract

The analysis of whole genomes has revealed specific geographical distribution of *Mycobacterium tuberculosis* (Mtb) strains across the globe suggestive of unique niche dependent adaptive mechanisms. We provide an important correlation of a genome‐based mutation to a molecular phenotype across two predominant clinical Mtb lineages of the Indian subcontinent. We have identified a distinct lineage specific mutation‐G247C, translating into an alanine‐proline conversion in the *pap*A2 gene of Indo‐oceanic lineage 1 (L1) Mtb strains, and restoration of cell wall sulfolipids by simple genetic complementation of *papA*2 from lineage 3 (L3) or from H37Rv (lineage 4‐L4) attributed the loss of this glycolipid to this specific mutation in Indo‐Oceanic L1 Mtb. The investigation of structure of Mtb PapA2 revealed a distinct nonribosomal peptide synthetase (NRPS) C domain conformation with an unconventional presence of a zinc binding motif. Surprisingly, the A83P mutation did not map to either the catalytic center in the N‐terminal subdomain or any of the substrate‐binding region of the protein. On the contrary, the inherent ability of mutant PapA2 to form insoluble aggregates and molecular simulations with the wild‐type/mutant (Wt/mut) PapA2 purports an important role for the surface associated 83rd residue in protein conformation. This study demonstrates the importance of a critical structural residue in the papA2 protein of Mtb and helps establish a link between observed genomic alteration and its molecular consequence in the successful human pathogen Mtb.

**Significance**

We demonstrate the effect of a unique SNP in *PapA2* gene of Indo‐oceanic *Mycobacterium tuberculosis* (Mtb) strains leading to the loss of sulfolipid from these strains. By X‐ray crystallographic analysis and molecular dynamics (MD) simulations, we show the importance of this residue in the global PapA2 structure. The presence of a Zn atom has not been reported before for this class of proteins. Here, we provide an important link between genomic alteration and its molecular consequence in Mtb highlighting one of the many adaptive mechanisms that have contributed to its success as a human pathogen. A high degree of identity with PapA1, 3, or 4 would help in interpreting the structure of these PapA proteins and other acyl transferases of other biological systems.

**Abbreviations**
Mtb: Mycobacterium tuberculosis
SNP: sinlge nucleotide polymorphism
PapA2: polyketide synthase associated protein A2
Mut: mutation
Wt: wild type
SL-1: sulfolipids-1

## 1 INTRODUCTION

*Mycobacterium tuberculosis* (Mtb) has the dubious distinction of being one of the most successful human pathogens by virtue of its extreme adaptability and survivability in the face of stress. Mtb being an intracellular pathogen has evolved to sense and manipulate the host to its advantage. Whole‐genome sequencing methods have classified Mtb strains across the globe into seven major lineages (L1‐L7) that have coevolved with its specific host population and environment.^1^

The Mtb cell wall is now recognized as a complex entity unique in its composition of complex polyketide lipids like trehalose dimycolate (TDM), SL/SL‐1 (sulfolipid), diacyl/polyacyl trehalose (DAT‐PAT), phthiocerol dimycocerosate (PDIM). This entity (cell wall) requires a finely tuned array of metabolic functions involving the biosynthesis, maturation, transport, and assembly of precursors from the cytoplasm to the exterior.^2^, ^3^, ^4^, ^5^ The ability of Mtb strains to alter their cell wall repertoire to effectively communicate with host cells, modulate immune signaling, and play a pivotal role in intracellular fitness is well recognized.^6^, ^7^, ^8^, ^9^, ^10^ Unique lipids like phenolic glycolipid (PGL) in Mtb strains are associated with downregulation of the inflammatory response and consequent hypervirulence of the strains.^11^, ^12^, ^13^ Interestingly, minor modifications like cylopropanation of mycolic acids leads to marked alterations in the host immune activation/suppression.^14^, ^15^ Mtb has evolved to manipulate the expression of its lipids as a counter for intracellular stress.^16^, ^17^

Sulfolipids represent Mtb‐specific lipids that have been the focus of research over the last several years.^18^, ^19^, ^20^ The presence of sulfolipids has been classically associated with virulence of mycobacteria.^21^, ^22^, ^23^, ^24^ Moreover, recent evidence has further corroborated their role in bacterial physiology. The biosynthetic pathway of mature sulfolipids in Mtb has been well characterized with the synthesis involving stepwise addition of four fatty acyl chains to sulfated trehalose by acyl transferases—PapA2 (Rv3820c), PapA1 (Rv3824c), and Chp1(Rv3822) coupled to export of the lipid to the outer cell wall by the transporters—Mmpl8 and Sap.^3^, ^25^, ^26^

In this study, we have employed whole‐genome–based analysis to pinpoint the molecular basis of loss of mature sulfolipid expression in the cell wall of the Indo‐Oceanic Mtb lineage (a subset of Mtb lineage 1). We demonstrate that a G247C SNP in the *papA*2 gene, encoding the first acyl transferase in sulfolipid biosynthesis, results in a detrimental modification of the alanine‐83 to proline. The expression of *papA*2 from H_37_Rv or N24 (lineage 3) was sufficient to restore mature sulfolipid in the cell walls of deficient strains establishing that this mutation is solely responsible for the loss of sulfolipid in these strains. By using X‐ray crystallography, we demonstrate that PapA2 attains a classical nonribosomal peptide synthase (NRPS) condensation (C) domain architecture with two subdomains arranged in V shape, each with coenzyme‐A–dependent acyltranferase (CAT) fold, two crossover points and catalytic center at the interface of two subdomains. The presence of a distinctive Zn finger motif in the N‐terminal region of Mtb PapA2 represents a unique modification of this protein from other known acyl transferases. By molecular dynamics (MD) simulation studies of mutant PapA2, we demonstrate that the A83P mutation induces significant misfolding of the protein resulting in global changes in protein conformation. Coupled to our inability to acquire soluble mutant protein from *Escherichia coli*, we provide evidence for an important role for the surface associated mutation in structural stability of Mtb PapA2 and SL‐1 biosynthesis.

## 2 MATERIAL AND METHODS

### 2.1 Bacterial cell cultures

*Mycobacterium tuberculosis* strains were grown in Middlebrook 7H9 (BD Biosciences, Gurgaon, India) media containing Albumin Dextrose Catalase (ADC; BD Biosciences, Gurgaon, India) at 37°C under shaking conditions unless stated otherwise. The Mtb clinical strains (Table S1) were a kind gift of Dr Sebastien Gagneux, Swiss TPH and part of the San Francisco collection.^1^ *E coli* was cultured as per standard procedures in LB broth or agar (BD Biosciences) with supplementation of kanamycin (50 μg/mL) or carbenicillin (100 μg/mL) when needed.

### 2.2 Analysis of lipids from Mtb

A quantity of 10 ml Mtb grown to the logarithmic phase in 7H9 media was supplemented with 1 µCi of ^14^C‐acetate (American Radiolabeled Chemicals, Inc, St. Louis, MO, USA) for 24 h at 37°C following which the polar and apolar lipids were isolated according to standard protocols.^27^ The extent of radiolabel incorporation was determined by using the TopCount NXT scintillation counter (PerkinElmer, Akron, OH, USA). Lipids equivalent to 10000 cpm for all the three strains were spotted on TLC silica gel 60 (Merck Millipore, Danvers, MA, USA) and eluted using solvents (A‐D) for apolar lipids and (D and E) for polar lipids.^27^ The TLCs were developed either on a photographic film or scanned using a GE Typhoon FLA 7000 phosphorimager system (GE Healthcare Bio‐Sciences, Dallas, TX, USA).

### 2.3 Cloning, expression, and purification of Mtb‐PapA2

For the mycobacterial expression of PapA2, the complete ORF of *papA*2 was PCR amplified from the genomic DNA of H37Rv (R), N24 (L3), or N73 (L1), and cloned into the mycobacterial expression vector pMV261 by using specific primers, A2F and A2R, to express the recombinant protein as a HA tagged fusion protein. For the expression in *E coli*, *papA*2 was amplified from the genomic DNA of H37Rv (wild‐type [Wt] PapA2) or N73 (mutant PapA2) using primers, A2expF and A2expR, cloned into pET28‐SMT3 vector to obtain the plasmid pVIP06. A list of primers is given in Table 1. The expression of recombinant protein following induction with  isopropyl‐β‐d‐thiogalactoside (IPTG; Himedia laboratories, Mumbai, India) was tested in the *E coli *strain C41(DE3) by SDS‐PAGE. For selenomethionine‐labeled protein (SeMet‐PapA2), cultures were grown at 25°C in selenoMet Dream Nutrient Mix (Molecular Dimensions, UK). A large‐scale purified protein was obtained from cultures induced with 0.1 mM IPTG for 24 hours at 18°C by using affinity columns (Ni‐NTA agarose, Qiagen, Germany). The protein was eluted with 250 mM of imidazole (Himedia laboratories, India), concentrated using Amicon Ultra Centrifugal Filters (Merck life sciences, Germany), and subjected to Ulp1 protease at 4°C for 16 hours for tag removal. Further purifications using  gel filtration (Superdex‐75 10/300gl‐GE Healthcare Life Sciences, UK) and anion exchange chromatography using a Resource Q column (GE Healthcare Life Sciences, UK) resulted in a > 90% pure protein. The expression of PapA2 was confirmed by immunoblotting with Tag‐specific antibody (ab18181‐HA/ab18184‐His, Abcam, UK).

**Table 1:** List of primers used in this study

S no	Primer	Sequence
1	A2F	GGGGATCCTACCCGTACGACGTGCCGGACTACGCCGTGTTTAGCATTACAACGCTCCGCGACTG
2	A2R	CCCAGGTCCTCCTCCGAGATCAGCTTCTGCTCAAGCTTTCATGTGCCTGGTTTAAGTGTC
3	A2expF	AGGGATCCTTTAGCATTACAACGCTCCGCGAC
4	A2expR	GCAAGCTTTCACGTGCCTGGTTTAAGTGTCGC

### 2.4 Sample preparation and MALDI‐TOF mass spectrometry

For MALDI‐TOF, 25 µg of PapA2 protein subjected to trypsin digestion was injected into a MALDI‐TOF/TOF 5800 (AB Sciex, USA) and the fragments were identified from SwissProt.

### 2.5 Protein crystallization

Sparse matrix crystallization trials of PapA2 (at 10 mg/mL) were carried out with a Crystal Screen HT (Hampton research, CA, USA) by hanging drop vapor diffusion technique 28 at 25°C. Initial diffraction experiments were performed using crystals obtained in 0.2 mol/L MgCl_2_.4H_2_O, 0.1 mol/L C_2_H_12_AsNaO_5 _3H_2_O, pH 6.5, 20% w/v PEG 8000 buffer. Following further optimization, the crystals were stored frozen with 30% w/v PEG 8000 as a cryoprotectant.

### 2.6 Data collection and processing

Diffraction data for native PapA2 and SeMet‐PapA2 crystals were collected at the European Synchrotron Radiation Facility (ESRF, Grenoble, France) on the beam line BM14. PapA2 and SeMet‐PapA2 crystals were diffracted up to 2.16 Å and 2.49 Å resolutions, respectively. Data obtained were indexed and scaled using the program HKL‐2000.^29^ The scaled intensities were converted into structure factors using the program TRUNCATE (DOI‐10.1107/S0567739478001114) as implemented in CCP4.^30^ The phase problem was solved using selenium as heavy atoms and by applying single anomalous dispersion (SAD) phasing procedure using the AutoSol wizard in PHENIX.^31^, ^32^ The structure of PapA2 was determined by molecular replacement^33^ using chain (B) of SeM‐PapA2 as a template in Phaser^34^ in PHENIX, refined as a rigid body followed by restraint refinement using phenix.refine.^35^ The model was built into the electron density map using the program COOT.^36^, ^37^ The program PyMOL (PyMOL Molecular Graphics System, Schrödinger, LLC) was used to visualize and analyze the model.

### 2.7 Molecular dynamics simulation

The crystal structure of papA2 was cleaned and prepared using Maestro (Schrödinger) (Maestro, version 9.8, Schrödinger, LLC, New York, NY, 2014). The prepared structure was selected for generation of mutation, A83P, using an Accelrys Discovery Studio visualizer (Discovery Studio. "version 2.5." Accelrys Inc: San Diego, CA (2009). Both the Wt and mutant PapA2 structures were taken for further refinement by MD simulations by GROMACS and OPLS‐all atom force field (DOI‐10.1021/ja9621760) using Wt papA2 as the starting structure. Both structures were used for separate MD simulations for 1 μs each. Each of the starting structure was placed in a cubic box solvated using TIP4P water representation (DOI‐10.1080/00268978500103111) (Table 2). The systems were neutralized using Na^+^ ions. The starting structures were subjected to energy minimization using the steepest descent method. Systems were simulated at 300K using the Nose‐Hoover T‐coupling (DOI‐10.1063/1.447334) and then later subjected to a Parrinello‐Rahman barostat (DOI‐10.1063/1.328693) for pressure coupling at 1 bar, before starting the production run. Electrostatic interactions were calculated using the particle mesh Ewald (PME) summation (DOI‐10.1063/1.464397).

**Table 2:** Details of MD simulation of Wt and mutant PapA2

Structure	No. of atoms	No. of solvent molecules	No. of protein atoms	No. of Na^+ ^ions	Simulation time
PapA2_wild	98650	91548	7088	14	1 μs
PapA2_mutant	98646	91540	7092	14	1 μs

## 3 RESULTS

### 3.1 Genome sequence analysis provides crucial insight into the loss of mature sulfolipid in lipid scaffold of lineage 1 Mtb strains

**Figure 1:**
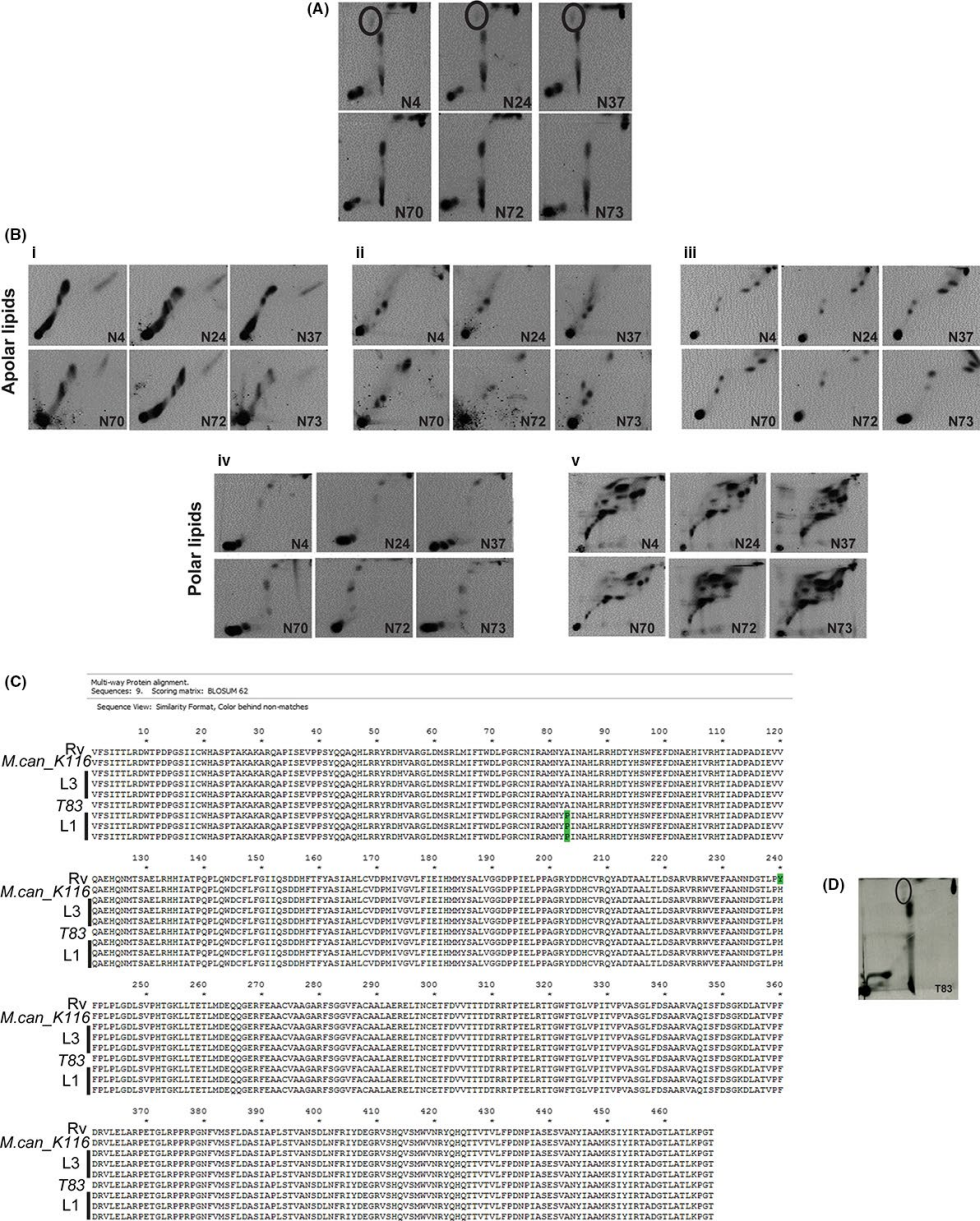
A nonsynonymous SNP in *PapA2* gene of Indo‐Oceanic Mtb strains is responsible for loss of mature SL‐1. A and B, Analysis of radiolabeled lipids by 2D TLC: A) Apolar lipids of ^14^C‐acetate–labeled Mtb Indo‐oceanic L1 (N73, N70, N72) and L3 (N24, N4, N37) were resolved in solvent D and visualized by radioimaging. The mature sulfolipid SL‐1 spot is encircled. B, Analysis of total lipids from ^14^C‐acetate–labeled cultures by 2D TLC in the different solvent systems (i—solvent A, ii—solvent B, iii—solvent C, iv—solvent D, and v—solvent E). The data from one representative analysis of N = 3 is shown. C, Comparison of PapA2 sequence of the reference Mtb strain (H_37_Rv), *M. canetti K116*, the three strains each of Indo‐Oceanic L1 (N73, N70, N72) and L3 (N24, N4, N37) and the L1 strain from Vietnam—T83. The mutated residue A83P is marked in green. D, Analysis of apolar lipids from Mtb T83 from ^14^C‐acetate–labeled logarithmic cultures resolved in solvent D is shown. The mature sulfolipid‐ SL‐1 spot is encircled. The solvents used are summarised in Table 3

Previous studies^38^, ^39^ have demonstrated the loss of sulfatides from Mtb strains of South India (Indo‐oceanic L1). In order to test if the differences can also be extended to the other predominant strain of the subcontinent—(L3) in northern India, we investigated the total cell wall associated lipid content of these two Mtb lineages. Figure 1 shows the 2D TLC lipid profiles of three strains, each from Mtb lineages 1 and 3.

**Table 3:** List of solvents used for analysis of Mtb lipids by 2D TLC

	Components	Ratio	No. of runs
Direction I			
System			
A	Pet ether/ethyl acetate	98:2	3
B	Pet ether/acetone	92:8	3
C	Chloroform/methanol	96:4	1
D	Chloroform/methanol/water	100:14:0.8	1
E	Chloroform/methanol/water	60:30:6	1
Direction II			
System			
A	Pet ether/acetone	98:2	1
B	Toluene/acetone	95:5	1
C	Toluene/acetone	80:20	1
D	Chloroform/acetone/methanol/water	50:60:2.3:3	1
E	Chloroform/acetic acid (glacial)/methanol/water	40:25:3:6	1

A uniform absence of SL‐1 from the apolar lipid fraction was the most distinct feature in all the three strains of Indo‐Oceanic L1 Mtb (Figure 1A). Most of the other apolar or polar lipids were consistent in both the lineages (Figure 1B‐i‐v). Similar loss of SL‐1 was also confirmed in lipid extracts of Mtb extracts without any tracer labeling (cold cultures, data not shown).

In an attempt to understand the molecular basis of this sulfolipid loss in (Indo oceanic L1), we resorted to genome sequence comparison with previously reported L3 strains and the reference strain H_37_Rv.^40^ A closer examination of the SNP list pointed toward a common mutation in the Indo‐Oceanic L1 Mtb genomes (pos. 428579); this G to C conversion resulted in conversion of the 83rd alanine of PapA2 (a polyketide‐associated acyl transferase involved in sulfolipid biosynthesis of Mtb) to proline (Figure 1C); a nontolerable mutation to protein function and structure (SIFT analysis). Interestingly, this mutation was not observed in *Mycobacterium canetti* or the other closely related L1 strain T83 (belonging to the Vietnam region) indicating specificity of this mutation to the Indian subcontinent (Figure 1C); consequently, T83 strain was capable of producing mature SL‐1 in the cell wall associated apolar lipid fraction (Figure 1D).

### 3.2 Transcomplementation of *papA*2 from L3 genome restores SL‐1 biosynthesis in representative L1 strain

**Figure 2:**
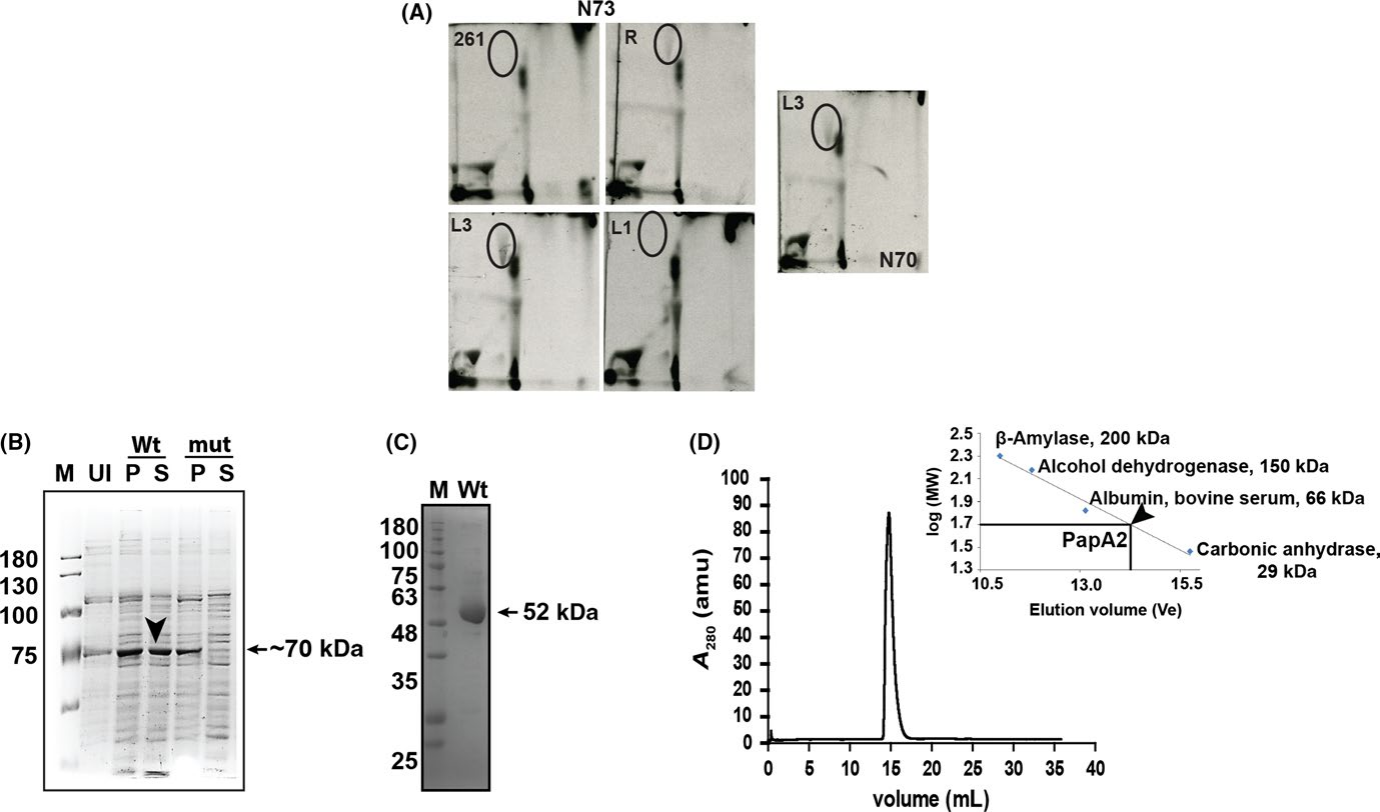
Functional complementation of SL‐1 in L1 Mtb by PapA2 from the L3/L4 Mtb strains. A, Analysis of apolar lipids from PapA2 expressing Indo‐Oceanic L1 Mtb strains. Lipids from ^14^C‐acetate–labeled cultures analyzed in solvent D by TLC. The SL‐1 spot is encircled. 261 refers to vector control—pMV261, R, L3, and L1—PapA2 from H_37_Rv, Mtb lineages 3 (N24) and 1 (N73), respectively. The apolar lipids from another Indo‐Oceanic L1 strain (N70) transformed with L3 PapA2 is also shown. The data from one representative analysis of N = 2 is shown. B, Expression profile of Wt‐PapA2 and A83P‐PapA2 was analyzed at indicated time points postinduction using 100 µM IPTG through SDS‐PAGE. Induction experiments were performed at 18°C. M is a protein ladder, Wt and mut represents wild‐type and A83P PapA2, U is uninduced protein sample, and P and S are pellet and soluble fraction of induced protein samples. The black arrow indicates the expression of Wt PapA2 in the soluble fraction. C, Analysis of the purified Wt PapA2 by SDS‐PAGE. D, Analysis of oligomeric state of the purified Wt PapA2 by gel exclusion chromatography. The elution profile of Wt papA2 is depicted; the elution volumes of standard proteins used for mass calculation are indicated in the inset graph

The presence of a deleterious nonsynonymous mutation, A83P, only in PapA2 allowed us to hypothesize its association with the absence of SL‐1 in Indo‐Oceanic lineage 1. In order to test this hypothesis, we ectopically expressed HA‐tagged *papA*2 gene from H_37_Rv or L3 in the Indo‐Oceanic L1 strain N73 (Figure 2A). We checked for the restoration of SL‐1 biosynthesis by 1D as well as 2D radiometric TLC. The restoration of SL‐1 was observed only when *papA*2 was expressed from H_37_Rv or L3, but not when *papA*2 of Indo‐Oceanic L1 or in case of vector control were used (Figure 2A). Similar restoration of SL‐1 in another Indo‐Oceanic L1 strain N70 explicitly confirms the causative role of A83P mutation in the loss of PapA2 function and consequent SL‐1 in this subset of L1 Mtb strains.

To test if this mutation affected the structural integrity of PapA2 or its function, we expressed both the Wt and mutant proteins in *E coli*. While we could obtain ~50% soluble protein expression of the Wt, the mutant protein partitioned to insoluble fractions in all conditions of culture and expression (Figure 2B) suggestive of a strong influence of the mutation on overall protein structure of PapA2. We resorted to a two‐step approach to confirm the role of this mutation in protein structure: (a) establish structure of the Wt protein and (b) understand the effect of A83P substitution by MD simulation. A significantly pure in excess of 90% of native PapA2 in the monomeric state (Figure 2C,D) was subjected to X‐ray crystallography for determination of structure after confirming the identity of the purified protein by MALDI‐TOF mass spectrometry (Figure S1).

### 3.3 PapA2 structural features display an unusual NRPS C domain architecture

The structure of PapA2 was determined at resolution of 2.16 Å using the phase calculated from anomalous diffraction of selenium as described in Methods (PDB ID‐ 6AEF). Details of data collection and data processing are summarized in Table 4.

**Table 4:** Crystallographic data collection and refinement statistics

Dataset	Native	SeMet
Data collection		
Space group	P2_1_2_1_2_1_	P2_1_2_1_2_1_
Cell dimension		
a, b, c (Å)	74.71, 100.7, 128.9	74.67, 97.49, 128.8
α, β, γ (°)	90°, 90°, 90°	90°, 90°, 90°
Resolution (Å)	50‐ 2.16 (2.25‐ 2.16)	50‐ 2.5 (2.54‐ 2.5)
Redundancy	6.5 (3.5)	8.7 (8.3)
R_merge_	9.5 (81.5)	11.7 (61.3)
CC (1/2) (%)	78.7	90.4
<I/σI>	19.7 (2.24)	22.09 (3.59)
Completeness (%)	99.5 (96.2)	96.2 (97.1)
Refinement		
Resolution (Å)	39.71‐2.16	
No. of reflections	52058	
R_work_/R_free_	0.1981/0.2315	
No. of atoms	14,660	
Protein	14,159	
Water	472	
Zn	2	
ACT	7	
TRS	20	
B factors	46.0	
Protein	32.1	
Water	36.8	
RMSD		
Bond lengths (Å)	0.005	
Bond angles (°)	0.711	

**Figure 3:**
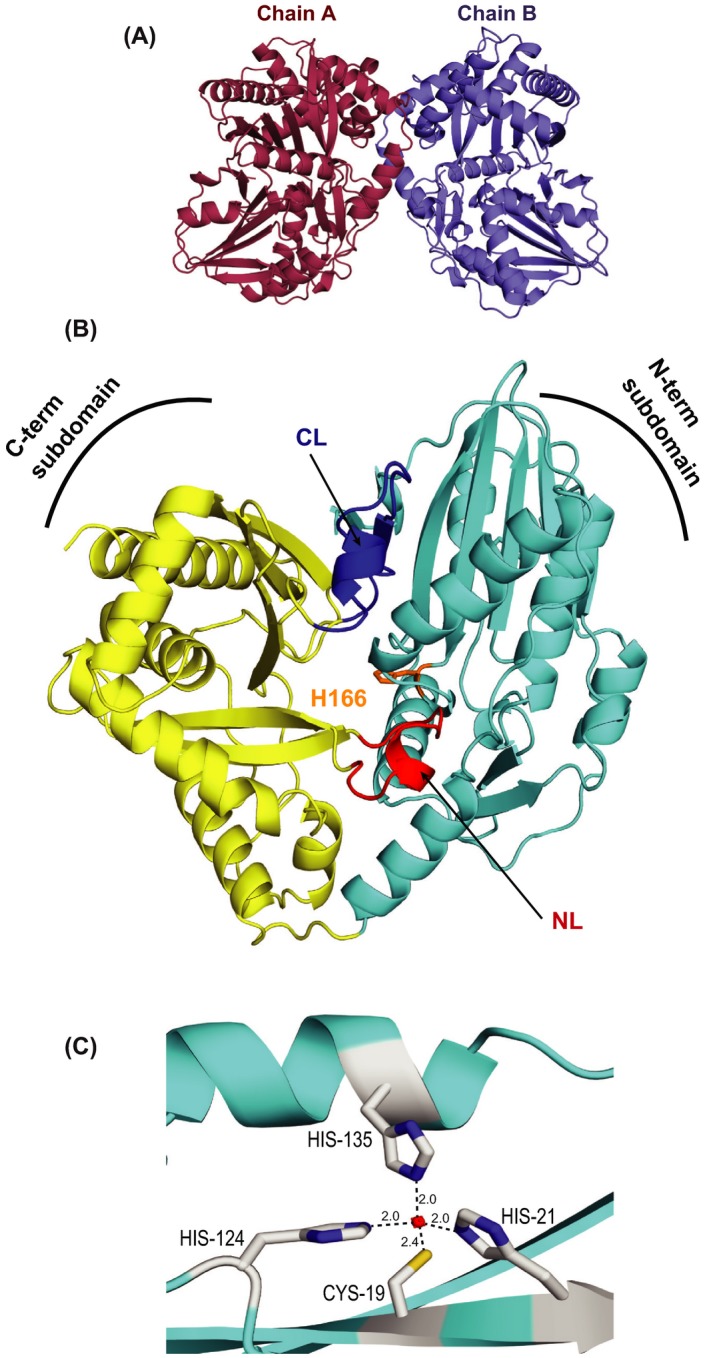
PapA2 portrays a two‐domain architecture. A, Asymmetric unit of the crystal contains two molecules of PapA2—chains A and B. B, The overall structure of PapA2. N‐terminal subdomain is cyan, C‐terminal subdomain is golden yellow, and N‐terminal and C‐terminal latches are shown in red (NL) and blue (CL) color, respectively. The location of active site H166 is shown in orange color. C, The putative Zn finger motif is shown—Zn atom (red circle) and coordinating residues (gray)

The asymmetric unit possess two molecules (Figure 3A). Each monomeric structure can be further described by dividing the protein into two subdomains: a N‐terminal subdomain (residues 2‐215) and a C‐terminal subdomain (residues 216‐459), each with a classical CAT fold comprising of a large β sheet flanked by alpha helices (Figure 3B). The core β sheet in the N‐terminal subdomain encompasses seven mixed‐type beta strands (parallel and antiparallel)—β1, β2, β3, β6, β7, β8, and β13—whereas the C‐terminal subdomain contains six mixed beta strands—β9, β10, β11, β12, β14, and β15 (Figure 3B). The two subdomains are connected by two crossover points—“latches,” wherein the C‐terminal subdomain extends back to the N‐terminal subdomain: (a) “N‐terminal latch”—residues 311‐323, forming helix α14 and (b) “C‐terminal latch”—residues 391‐411, including helix α18 followed by a beta strand β13 (Figure 3B). Most importantly, structure analysis indicated the presence of a unique Zn binding motif (ZnF) in the N‐terminal subdomain comprising of residues—C19, H21, H124, H135 (Figure 3C). The presence of Zn in the native protein was further confirmed through ICP‐MS

### 3.4 The interface region of PapA2 possesses substrate binding sites

**Figure 4:**
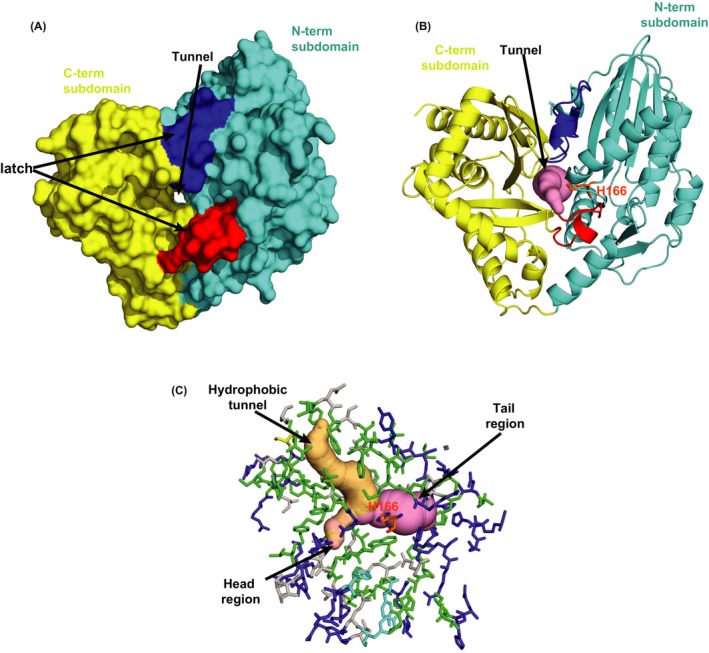
The Interface region of two subdomains harbors a tunnel for putative substrate binding. A and B, Solid surface (A) and cartoon presentation (B) of PapA2 protein structure. The tunnel (pink) is seen at the center of interface regions of two subdomains—N‐terminal subdomain (cyan) and C‐terminal subdomain (golden yellow). The two crossover points or latches are shown in red and blue. C, Stick presentation of the interface region along with pseudotunnels displaying void regions in the protein. The tunnel (pink) and hydrophobic tunnel (light orange) are marked. Residues lining the interface region are color coded based on their polarity; hydrophobic (green), charged (tv blue), and polar (gray). The active site is shown in orange

Solid surface analysis revealed the presence of a solvent accessible tunnel at the interface of two subdomains (Figure 4A). The placement of pseudotunnel at the interface of two subdomains using Caver program 41 and metapocket server 42 showed H166, the catalytic center,^43^ in the middle of the tunnel indicating a potential substrate binding site(s). This ~25 Å long tunnel originated close to the “N‐terminal latch” and ended before helix 3 with an access to His166 from both ends will henceforth be referred to as the “tunnel” (Figure 4B).

Further detailed analysis revealed a well‐organized arrangement of residues with distinct polarity at the “interface” tunnel with a dense population of positively charged arginine residues in the tail region of the tunnel and a more diverse distribution of residue polarity in the head region of the tunnel (Figure 4C). Interestingly, another cavity enriched in hydrophobic residues at one end (the “hydrophobic tunnel”) intersects with the tunnel in close proximity to the catalytic H166 suggesting a putative binding site for the large hydrophobic acyl chain of the donor substrate.

### 3.5 Molecular docking provides evidence for a unique substrate approach to PapA2

We further investigated the interface region by molecular docking using the acceptor and donor substrates. We reasoned that the catalytic H166 should be in close proximity of the acylation site of the substrate for efficient catalysis.

**Figure 5:**
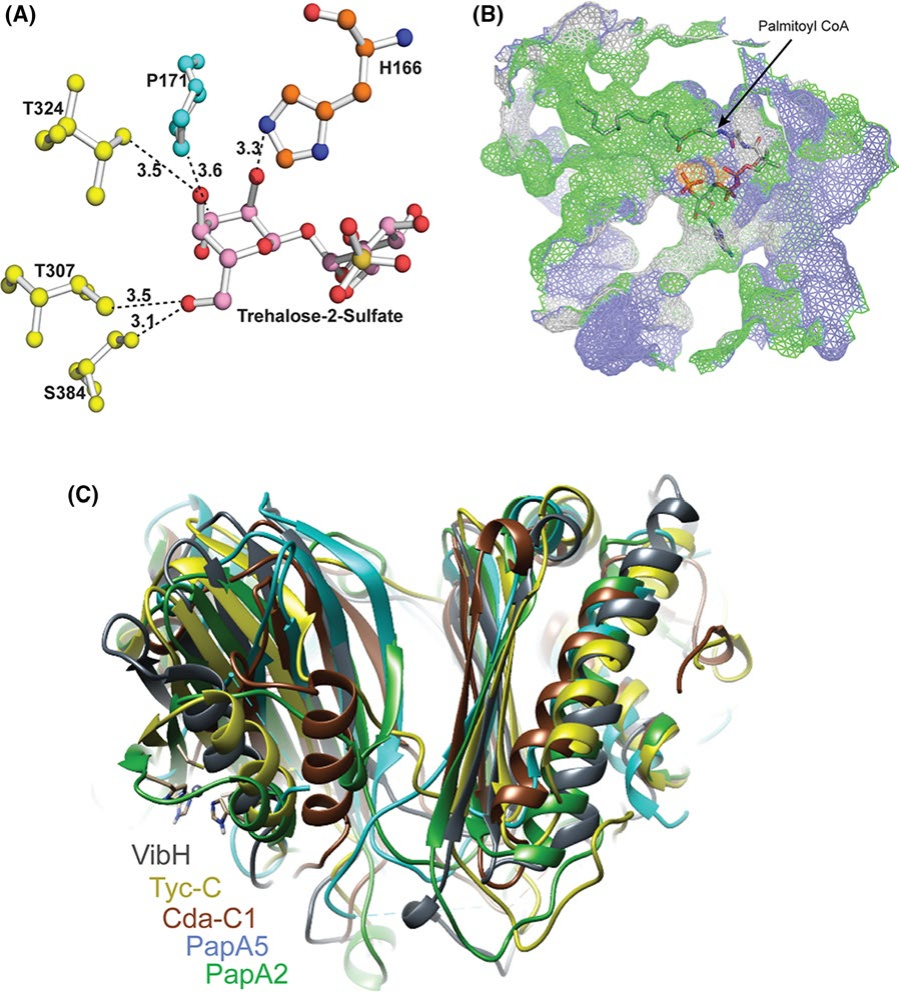
Detailed analysis of the acceptor and donor substrate binding sites. A, Ball and stick model of the substrate binding sites. The 2'‐OH group of acceptor substrate is in proximity to ε nitrogen of H166 of condensation motif, with P171, T307, T324, and S384 being additional residues in proximity to the acceptor substrate. B, Mesh surface representation shows the donor substrate conformer with the acyl component residing in a hydrophobic tunnel and the CoA component in close proximity of the tail region of the solvent accessible tunnel. C, Superposition of PapA2 with other stand‐alone C domain structures revealed a distinct NRPS C domain architecture

Using this as a reference, we selected the conformer that positioned the 2'‐OH of trehalose‐2‐sulfate in the proximity of H166 and also identified four residues—P171, T307, T324, and‐ S384—in apposition of the ligand (Figure 5A). Similar docking studies also clearly placed the long acyl chain of palmitoyl CoA (donor substrate) in the hydrophobic tunnel and the CoA moiety in proximity to the tail region of the open tunnel (Figure 5B) revealing a putative unique bidirectional substrate approach to the catalytic center of PapA2—access of the acceptor substrate from the head region of the open tunnel and entry of donor substrate from the tail region during the acylation reaction.

In an effort to define the substrate binding region, we superimposed PapA2 with previously reported structures of other C domain proteins—the Mtb polyketide‐associated protein (PapA5, Rv 2939),^44^ condensation (C) domain of calcium‐dependent antibiotic synthetase (CDA‐C1),^45^ tyrocidine synthetase III (TycC),^46^ surfactin A synthetase C (SrfC), 47 and vibriobactin synthase (VibH).^48^ Although, we observed an overall conservation of architecture of the proteins with the catalytic histidine residing in the subdomain interface region, and conserved positioning of secondary elements in the C‐terminal subdomain, considerable conformational differences were observed in the N‐terminal subdomain (Figure 5C). Previous studies have identified the key acceptor substrate determinants for some of these proteins by mutagenesis.^45^, ^48^, ^49^ Our docking studies identified important residues of the head region of the solvent accessible tunnel—P171, T307, T324, S384 residing in close proximity of the acceptor substrate (Figure 5A). Mapping with the other C domain proteins revealed three of four residues of Mtb PapA2 (P171, T324 and S384) as positional equivalents of acceptor substrate determinants in VibH (G131 and N335) or CDA‐C1 (G162, S309), or PapA5 (G129) (Table 5).

**Table 5:** Molecular determinants for acceptor substrate from various C domain proteins

VibH	Cda‐C1	PapA5	PapA2
G131	G162	G129	P171
—	S309	—	T324
N335	—	—	S384
H126	H157	H124	H166
D130	D171	D128	D170

### 3.6 MD simulations suggest putative local and global changes in A83P mutant PapA2

**Figure 6:**
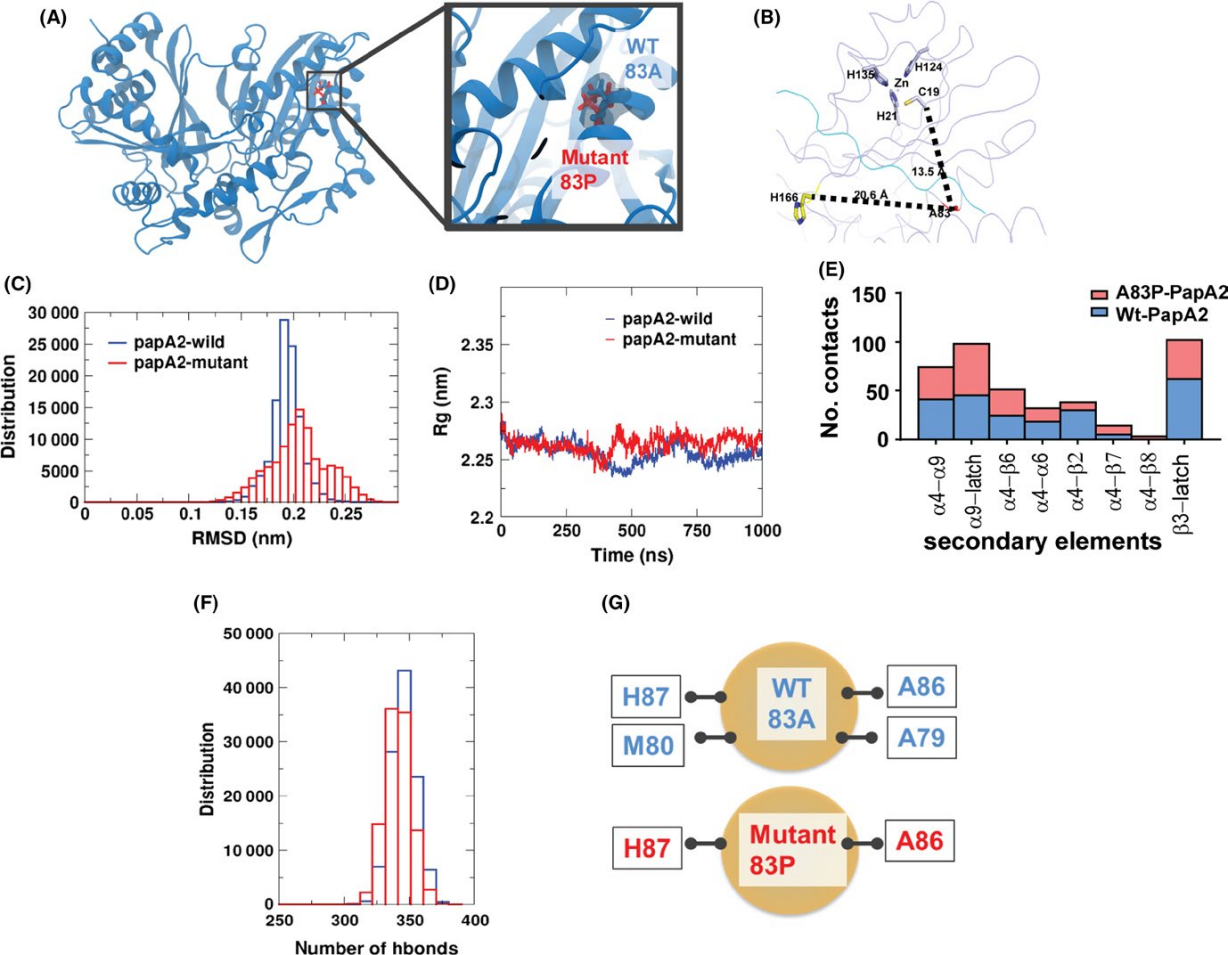
Molecular dynamics simulation reveals major alterations in the global fold of mutant PapA2. A, Representation of the models (Wt and mutant PapA2) structures used for MD simulation studies. B, Distance of the A83P mutation from the H166 catalytic residue and the Zn finger motif of PapA2. C, Graphical representation of the RMSD changes in Wt and mutant PapA2. D, Analysis of changes in the total size (radius of gyration [Rg]) of PapA2 protein on account of the A83P mutation. E, Analysis of numbers of local contacts of the 83rd residue in Wt (blue) and A83P (red)PapA2. The local secondary elements are represented on the x‐axis. F and G, Analysis of total hydrogen bond interactions (F) and hydrogen bonds interacting with the 83rd residue (G) in the Wt (blue) and A83P (red) PapA2.

Molecular dyanmics simulations have been extensively used in the past to understand intrinsic dynamics behavior for various proteins. Together with crystallographic data, this complementary approach captures dynamics and structural insights of protein conformational state. To generate the starting structure of mutant, we modeled the A83P residue site of PapA2 structure (Figure 6A). Interestingly, the mutation mapped to the protein surface (α4 helix) distal to the other functional sites—the ZnF motif and catalytic motif (Figure 6B). Two independent MD simulations of the Wt and mutant PapA2 were performed for a cumulative 1 μs simulation time.

Comparison of Wt and mutant trajectories revealed global structural rearrangements in the mutant protein (Figure 6C‐D). The order parameters, RMSD and Rg, capture the mobility and overall compactness of the protein, respectively. Simulations with the mutant PapA2 protein revealed protein ensembles exhibiting >0.2 nm RMSD and an increase in Rg at ~400 ns in comparison to the Wt protein, hinting at an increase in protein mobility and global changes as a result of the mutation. Interestingly, a significant increase in the flexibility of the C‐terminal latch (> 0.5 nm) in A83P‐PapA2 was again supportive of the distal effects of the A83P mutation on PapA2 flexibility and overall the global fold of the protein.

Concomitant to global changes, we also monitored local dynamical changes induced by the mutant residue. Analysis of neighboring residues that directly contact residue 83 of PapA2 identified major alterations. Local contacts were significantly reduced for the α4‐α6, α4‐α2 and β3‐latch regions with increase in contacts of the α4‐β7 region as a result of the proline mutation in mutant PapA2 (Figure 6E). In addition, while A83 bonded with H87, M80, A86, and A79 via hydrogen bonds, analysis of the P83 interactions reduced the local network to only two bonds H87, and A86 (Figure 6F,G). These observations further support the local destabilization of noncovalent interactions as nucleation sites for global protein unfolding and an alteration of overall protein stability.

**Figure 7:**
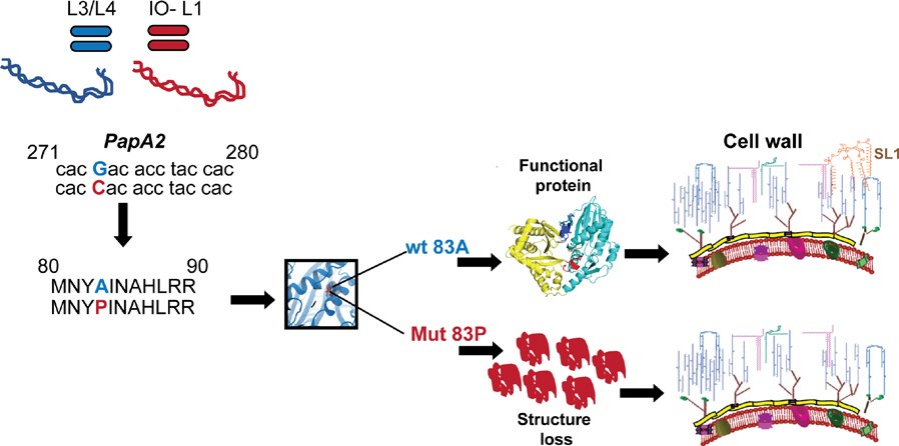
A model for A83P‐mediated loss of SL1 in Mtb

A putative model for the mutation‐associated loss of PapA2 function is represented as Figure 7.

## 4 DISCUSSION

The mycobacterial cell wall is one of the most complex chemical entities replete in lipid and carbohydrate moieties not found elsewhere in biological systems apart from actinomycetes. The intricate molecular mechanisms for the biosynthesis, maintenance, and plasticity is yet not well understood; more so, the adaptability in the face of physiological stress and environmental pressures. Given the long evolution of Mtb, its adaptation with the human host in the context of its genomic and molecular repertoire is now being recognized as an important factor for the successful survival. Recent molecular evidences have pointed out lineage specific variations in Mtb, culminating as a result of both host driven and environmental cues, correlating with the inflammatory potential of the pathogen.^1^, ^50^, ^51^ Often, a clear correlation between the observed lineage‐specific genetic attributes and its molecular/phenotypic characteristics or vice versa remains poorly characterized.

In an effort to understand the molecular basis for loss of sulfolipid in clinical strains of Mtb (a small subset of Mtb lineage 1), we identified a single SNP in the deficient strains mapping to the PapA2 coding region of the genome. This resultant conversion of the 83rd alanine to proline of PapA2, one of the primary enzymes of the mycobacterial sulfolipid biosynthetic machinery manifested as a compromise in protein stability and folding, significant enough to prevent our attempts to obtain purified, soluble mutant protein even by chemical chaperones. This global defect in the fold of the mutant protein was further supported by MD simulation studies and emphasizes a crucial role of the A83 residue in structural integrity of PapA2.

In agreement with the structure of other C domain multidomain peptide synthase proteins of NRPS,^44^, ^45^, ^47^, ^48^ PapA2 also conformed to a typical V‐shaped two subdomain containing architecture with two latch components and catalytic histidine at the interface of two subdomains. The distinct presence of (a) a solvent accessible tunnel in close apposition with the catalytic site and (b) hydrophobic tunnel implies a dual substrate approach strategy—an acceptor substrate (T2S) approaching from the head region and the donor acyl CoA substrate from the tail region (acyl group residing in the hydrophobic‐rich region) and the CoA in close proximity of the tail region. Remarkably, the A83P mutation mapped to the protein surface away from the catalytic and zinc finger containing regions (20 Ǻ and 13 Ǻ respectively) of L1 PapA2. Our conclusion of the mutation site distant from the active site deteriorates protein structure and function is in agreement with the reports on the effect of distant mutation in the proteins.^52^, ^53^ Interestingly, the occurrence of hydrophobic residues, such as Ala or Val, in this position is highly conserved in all the members of the PapA proteins of Mtb suggestive of the importance of this Ala residue in the function of this protein family (Figure S2).

The most interesting aspect of the structure was the identification of a putative Zn finger motif in PapA2. The presence of this motif implies the possibility of protein‐protein/protein‐DNA interactions^54^, ^55^, ^56^ unique to any of the acyl transferase proteins identified. Interestingly, despite considerable sequence identity among other members of the Mtb PapA family of proteins (PapA1, PapA3, and PapA4), this consensus motif is restricted to PapA2 signifying the importance of this component in PapA2 function.

While PapA2 is involved in the first acylation of trehalose sulfate, the related acyl transferase‐PapA1 catalyzes the addition of a second acyl chain to this monoacylated sulfated trehalose. We identified a long hydrophobic tunnel that could house the acyl CoA donor necessary for PapA2 function. However, PapA1 function requires two acyl chains to be fitted in close proximity of each other (acyl group of monoacylated T2S and the second fatty acyl CoA). Given the Zn finger motif in PapA2, it is logical to assume a direct interaction between PapA2 and PapA1 in order to facilitate the dual acyl chain transfer. In fact, previous studies have also proposed a similar multicomponent “scaffolding” model for the biosynthesis of SL‐1, which require biosynthetic components in close proximity to each other.^3^ Identifying the interactions between the PapA proteins of mycobacteria would provide conclusive evidence of such novel functions associated with cell wall lipid assembly in Mtb.

With sulfolipid limited to members of the Mtb complex despite the presence of a papA2 homolog (with close to 50% homology) in other nontuberculous mycobacteria, an important role for this lipid in bacterial physiology can be envisaged. The exclusive presence of this consensus Zn binding motif in PapA2 of Mtb complex (absent even in the related pathogenic *Mycobacterium marinum)* again hints at an important role for this motif in protein function. However, the role of sulfolipids in Mtb pathogenesis is confusing. Classical studies have linked the expression of sulfolipids to the degree of virulence associated with Mtb.^38^ However, Rousseau et al^57^ have clearly unlinked the presence of sulfolipid with virulence in H_37_Rv. While the importance of sulfolipid in host‐pathogen cross talk can be envisaged given its localization to outer most layer of the cell, it has also been implicated in inhibition of phagolysosome fusion^21^ and modulating the proinflammatory response.^22^, ^23^ Alternatively, by functioning as a sink to buffer changes in propionyl CoA content, SL can contribute to metabolic reshuffling during in vivo growth.^16^, ^58^, ^59^ In contrast, mutants of H_37_Rv that lack sulfolipids viz. Δpks2, Δmmpl8 have not shown any defect in *in vivo* growth in mice models of infection.^7^, ^60^ Given the pleomorphic importance of PGL in Mtb virulence and its dependence on the strain genotype, it is plausible to expect that this specific loss of sulfolipid‐1 in the Indo‐Oceanic L1 strains specifically is an adaptive mechanism for the fine‐tuned balance of infection by these strains in the specific human population/environment. A careful elucidation of the importance of SL‐1 in mycobacterial immunopathogenesis in the context of specific lineages would aid resolve this conundrum. Our phenotype‐genotype correlation of a novel SNP resulting in the loss of a major surface glycolipid in a specific subset of Mtb provides an excellent platform to address specific adaptive mechanisms employed by a very successful human pathogen.

## CONFLICT OF INTEREST

The authors do not have any competing interests.

## AUTHORS CONTRIBUTIONS

VR conceptualized the work; VP, VR, SC, AB, and GB designed the work. VR and AB carried out the mycobacterial lipid work. VP and AM carried out protein expression and purification. VP and SC performed crystallization, structure determination, and structure analysis. NJ performed the molecular simulations; VP and NJ analyzed the molecular simulation results. VP, LT, VR, and SC contributed to the manuscript preparation.

## Supporting information

 ; Click here for additional data file.

 ; Click here for additional data file.

 ; Click here for additional data file.

 ; Click here for additional data file.

 ; Click here for additional data file.

 ; Click here for additional data file.
