# Supplementary figures and images for: A novel mutation alters the stability of PapA2 resulting in the complete abrogation of sulfolipids in clinical mycobacterial strains
Source: FASEB Bioadv. 2019 Apr 10;1(5):306–19. doi: 10.1096/fba.2018-00039 (PMC6996325; doi:10.1096/fba.2018-00039)

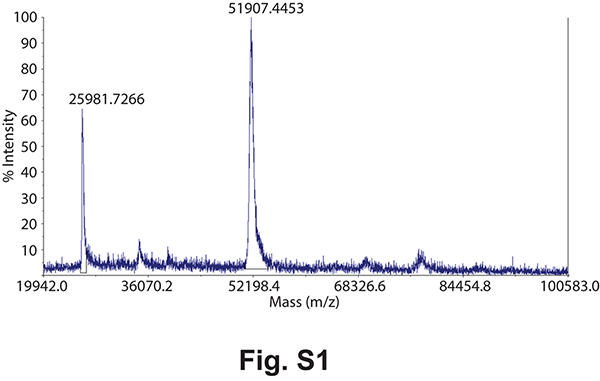

Supplement: Supplementary file 1 — ; [file FBA2-1-306-s001.png]

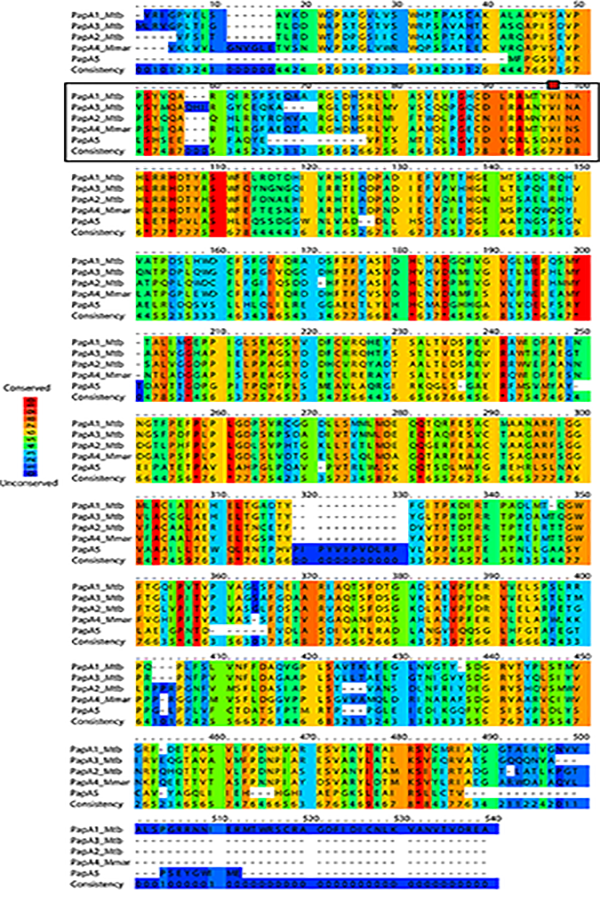

Supplement: Supplementary file 2 — ; [file FBA2-1-306-s002.png]
