# Supplementary material for: A novel mutation alters the stability of PapA2 resulting in the complete abrogation of sulfolipids in clinical mycobacterial strains
Source: FASEB Bioadv. 2019 Apr 10;1(5):306–19. doi: 10.1096/fba.2018-00039 (PMC6996325; doi:10.1096/fba.2018-00039)
Supplement: Supplementary file 3 — ; [file FBA2-1-306-s003.docx]

**Table S1. Summary of Mtb strains used in this study**

| Strain name | Lineage | Origin |
| --- | --- | --- |
| N70 | 1 | San Francisco |
| N72 | 1 | San Francisco |
| N73 | 1 | San Francisco |
| T83 | 1 | San Francisco |
| N04 | 3 | San Francisco |
| N24 | 3 | San Francisco |
| N37 | 3 | San Francisco |
| Erdman | 4 | Jeff Cox, USA |
