# Supplementary material for: A novel mutation alters the stability of PapA2 resulting in the complete abrogation of sulfolipids in clinical mycobacterial strains
Source: FASEB Bioadv. 2019 Apr 10;1(5):306–19. doi: 10.1096/fba.2018-00039 (PMC6996325; doi:10.1096/fba.2018-00039)
Supplement: Supplementary file 4 — ; [file FBA2-1-306-s004.docx]

**Table 2 Crystallographic data collection and refinement statistics**

| Dataset | Native | SeMet |
| --- | --- | --- |
| **Data collection** |  |  |
| Space group | P2_1_2_1_2_1_ | P2_1_2_1_2_1_ |
| Cell dimension |  |  |
| a, b, c (Å) | 74.71, 100.7, 128.9 | 74.67, 97.49, 128.8 |
| α, β, γ (˚) | 90°, 90°, 90° | 90°, 90°, 90° |
| Resolution (Å) | 50- 2.16 (2.25- 2.16) | 50- 2.5 (2.54- 2.5) |
| Redundancy | 6.5 (3.5) | 8.7 (8.3) |
| R_merge_ | 9.5 (81.5) | 11.7 (61.3) |
| CC (1/2) (%) | 78.7 | 90.4 |
| <I/σI> | 19.7 (2.24) | 22.09 (3.59) |
| Completeness (%) | 99.5 (96.2) | 96.2 (97.1) |
| **Refinement** |  |  |
| Resolution (Å) | 39.71-2.16 |  |
| No. of reflections | 52058 |  |
| R_work_/R_free_ | 0.1981/0.2315 |  |
| No. of atoms | 14,660 |  |
| Protein | 14,159 |  |
| Water | 472 |  |
| Zn | 2 |  |
| ACT | 7 |  |
| TRS | 20 |  |
| B factors | 46.0 |  |
| Protein | 32.1 |  |
| Water | 36.8 |  |
| RMSD |  |  |
| Bond lengths (Å) | 0.005 |  |
| Bond angles (˚) | 0.711 |  |
