# Supplementary material for: A novel mutation alters the stability of PapA2 resulting in the complete abrogation of sulfolipids in clinical mycobacterial strains
Source: FASEB Bioadv. 2019 Apr 10;1(5):306–19. doi: 10.1096/fba.2018-00039 (PMC6996325; doi:10.1096/fba.2018-00039)
Supplement: Supplementary file 5 — ; [file FBA2-1-306-s005.docx]

**Table 3 Molecular determinants for acceptor substrate from various C domain proteins**

| VibH | Cda-C1 | PapA5 | PapA2 |
| --- | --- | --- | --- |
| G131 | G162 | G129 | P171 |
| - | S309 | - | T324 |
| N335 | - | - | S384 |
| H126 | H157 | H124 | H166 |
| D130 | D171 | D128 | D170 |
