# Supplementary material for: A novel mutation alters the stability of PapA2 resulting in the complete abrogation of sulfolipids in clinical mycobacterial strains
Source: FASEB Bioadv. 2019 Apr 10;1(5):306–19. doi: 10.1096/fba.2018-00039 (PMC6996325; doi:10.1096/fba.2018-00039)
Supplement: Supplementary file 6 — ; [file FBA2-1-306-s006.docx]

**Table 4: Details of MD simulation of Wt and mutant PapA2**

| **Structure** | **No. of atoms** | **No. of solvent molecules** | **No. of protein atoms** | **No. of Na^+^ ions** | **Simulation time** |
| --- | --- | --- | --- | --- | --- |
| PapA2_wild | 98650 | 91548 | 7088 | 14 | 1 μs |
| PapA2_mutant | 98646 | 91540 | 7092 | 14 | 1 μs |
